# Sight-over-sound judgments of music performances are replicable effects with limited interpretability

**DOI:** 10.1371/journal.pone.0202075

**Published:** 2018-09-05

**Authors:** Samuel A. Mehr, Daniel A. Scannell, Ellen Winner

**Affiliations:** 1 Department of Psychology, Harvard University, Cambridge, Massachusetts, United States of America; 2 Data Science Initiative, Harvard University, Cambridge, Massachusetts, United States of America; 3 Department of Psychology, Boston College, Chestnut Hill, Massachusetts, United States of America; Universidad de Salamanca, SPAIN

## Abstract

Virtuosi impress audiences with their musical expressivity and with their theatrical flair. How do listeners use this auditory and visual information to judge performance quality? Both musicians and laypeople report a belief that sound should trump sight in the judgment of music performance, but surprisingly, their actual judgments reflect the opposite pattern. In a recent study, when presented with 6-second videos of music competition performers, listeners accurately guessed the winners only when the videos were muted. Here, we successfully replicate this finding in a highly-powered sample but then demonstrate that the sight-over-sound effect holds only under limited conditions. When using different videos from comparable performances, in a forced-choice task, listeners' judgments were at or below chance. And when differences in performance quality were made clearer, listeners' judgments were most accurate when they could hear the music—without audio, performance was at chance. Sight therefore does not necessarily trump sound in the judgment of music performance.

## Introduction

Music performances are inherently multimodal. That musicians entertain their listeners both with their musical expressivity and their theatrical flair provides a simple explanation for the widespread popularity of live concerts, even when the internet makes audio recordings of performances more accessible than ever. This multimodality presents issues, however, for evaluating the quality of music performances, because visual information—including the performer's sex, attractiveness, movement, and so on— could confound listeners' ability to make judgments of the quality of the *music* being performed. This would be consistent with findings demonstrating visual information's powerful effects on value judgments in a wide variety of commonsense domains. For instance, we are warned not to judge a book by its cover; department stores lay out their wares in an inviting, attractive fashion; and when selling one's house, a fresh coat of paint is advisable.

Does the visual information present in a music performance affect judgments of the quality of that performance? A widely publicized paper [1] reported that although both layperson and musician participants predicted that *sound* would most strongly influence their judgments, in fact, participants were best able to judge musical talent when viewing *silent* clips of performers rather than when viewing the same clips with audio or listening to the audio of those clips without any video. Further, the level of musical expertise of the listeners had no effect on judgment accuracy.

These findings included a number of internal replications, in an effort to determine what features of performance videos were driving this counterintuitive effect. For instance, when participants watched silent “outline” versions of the videos that had been processed to isolate the available visual information to motion alone, participants still accurately judged musical talent. Additional experiments asked participants not to choose the winning performer from silent videos, but rather to rate the performances on a variety of dimensions (e.g., creativity, involvement, motivation, passion). Here too listener ratings accurately reflected musical talent. These and other internal replications used the same set of source videos, however; thus, the degree to which the findings generalize to other musical performances is unknown.

Given both the well-known issue of publication biases [2] and questions of reproducibility in psychological science [3], counter-intuitive findings such as these call for replication and extension by independent researchers, and with broader stimulus sets. Here, we aimed to determine first whether the sight-over-sound findings reported in [1] are robust, and second, whether and how the findings generalize to other performance videos (whether from the same competitions or different ones). The former question is a practical one and simply determines the reliability of the original finding; we test it via a highly-powered direct replication (Experiment 1). The latter question is of psychological interest, as it helps to determine the scope of interpretation justified by the original findings; we test it via two highly-powered conceptual replications (Experiments 2 and 3).

## Experiment 1

### Method

We conducted a high-power replication designed to match the original experiments (Experiments 3 and 5 in [1]) as closely as possible. In the original experiments, participants listened to the three finalists in each of ten classical music competitions and judged which one of the three won the competition. We used nine of the original ten triads of videos. Rather than recruit a group of expert and a group of non-expert musicians, as in [1], we used a continuous measure of musical expertise (i.e., a test of auditory skills) in a single cohort.

#### Statistical power

We used original data reported in [1] (Exp. 3) provided by the author to conduct a power analysis prior to data collection. In the visual-only condition of that experiment, participants identified the winner 46.4% of the time, an effect of size *d* = .58 over chance level. We followed advice for replication with 2.5 times the sample size (*n* = 125 per condition) [4], achieving power greater than .999 to detect the original effect (power estimates conducted in G*Power [5]).

#### Participants

Both the Boston College and Harvard University IRBs approved this research. We recruited participants from Harvard University and Boston College's online subject participation databases. They viewed and/or listened to stimuli via Qualtrics, where they also provided their responses. Recruitment continued until each of the three conditions had reached the target sample size of 125; thus, analyses were performed on 375 participants (age: *M* = 21 years, *SD* = 5.1, range: [16, 65]; sex: 155 male, 199 female, 21 chose not to report).

#### Evaluation of musical expertise

The Musical Ear Test is used to assess musical ability and takes approximately 20 minutes to complete [6]. The test consists of 52 pairs of audio containing melodic phrases and 52 pairs of audio containing rhythmic phrases; for each pair, the participant is asked whether the two members of a pair of phrases are the same or different. The test has high internal consistency and clearly distinguishes between professional musicians and non-musicians, with musicians scoring about 2 SD higher than non-musicians, on average [6].

#### Competition videos

Competition videos were gathered from publicly available videos on YouTube, using links and clip times provided by the author of the original report. Detail on the methods of selecting these videos are available in the Supporting Information in [1] (see pp. 2–3).

#### Procedure

Participants first took the Musical Ear Test, using the instructions and materials provided by the test authors [6]. Next, participants were randomly assigned to the audio-only, visual-only, or audiovisual condition, and were told that they would be listening, watching, or listening and watching (respectively) to triads of clips of performers competing in international music competitions. In the audio-only condition, participants were presented only the audio from each clip of competition. In the visual-only condition, participants were presented only the video without audio. In the audiovisual condition, participants were presented with the video and audio together. The order of presentation of winning vs. non-winning performers for each triad was randomly sorted; the order of triads was determined randomly and was then fixed across participants. Each triad was presented once, and participants were then asked via online prompt which of the three clips was performed by the winner. Participants were asked to wear headphones.

### Results

First, we asked whether performance was highest in the visual-only condition, the main effect in [1], Exp. 3. Performance in the audio-only condition was significantly below chance (percent correct, *M* = 29.7, *SD* = 14.6, 95% CI: [27.1, 32.3]; *t*(124) = -2.79, *p* = .006) and performance in the audiovisual condition was not significantly different than chance (*M* = 36.4, *SD* = 18.6, 95% CI: [33.1, 39.7]; *t*(124) = 1.81, *p* = .072). In contrast, in the visual-only condition, performance was significantly above chance (*M* = 38.7, *SD* = 16.8, 95% CI: [35.7, 41.6], *d* = 0.32; *t*(124) = 3.55, *p* < .001); between-subjects, this level of performance was significantly higher than the two other conditions (*t*(373) = 3.04; *p* = .003). Thus, when we tested participants’ interpretation of 9 of the 10 clips originally used in[1], the replication was successful. As in [1], item analyses revealed that these effects were consistent across trials.

We then proceeded with two analyses examining whether musical expertise was related to performance on the main task. First, we used ordinal logistic regression to test the relation between Musical Ear Test scores and accuracy on the 9 test trials. No effects were found in any condition (*p*s > .3), demonstrating that auditory skills were unrelated to higher performance in identifying the competition winners. This provides a conceptual replication of previously reported effects [1], where separately recruited professional musicians performed comparably to non-musicians. To replicate this result more directly, we next restricted the sample to those participants performing in the top decile on the Musical Ear Test, or greater than 79.8% correct. This cutoff is higher than the minimum value reported in previous research with professional musicians [6], Exp. 3. Even with the resulting reduction in sample sizes (audio only: *n* = 19; visual-plus-audio: *n* = 13; visual only: *n* = 9) the main effect held. Performance in the visual-only condition was significantly above chance (percent correct, *M* = 42.0, *SD* = 9.26, 95% CI: [34.9, 49.1], *d* = 0.93; *t*(8) = 2.80, *p* = .023) but performance in the other two conditions was at chance (audio-only: *M* = 29.8, *SD* = 13.4, 95% CI: [23.4, 36.3]; *t*(18) = 1.14, *p* = .27; visual-plus-audio: *M* = 35.0, *SD* = 15.6, 95% CI: [25.6, 44.5]; *t*(12) = 0.39, *p* = .70); performance in the visual-only condition was significantly higher than the other two conditions (*t*(20.0) = 2.51, *p* = .02; Satterthwaite's *t*-test).

### Interim discussion

This highly-powered direct replication demonstrated that the main findings from [1] are reliable. With nearly-identical materials, the same pattern of results emerged: (1) participants of all musical abilities are able to identify the winners of music competitions when provided *only* with visual information; (2) they are unable to do so when provided with either audio-only or audiovisual information; and (3) participants' auditory skill level is unrelated to their ability to identify high-quality performers, regardless of the medium in which they are presented.

We next aimed to determine the generality of this finding via two conceptual replication attempts. In Experiment 2, we used a set of performances chosen from a mix of the same musicians, the same competitions, or both, and also simplified the task by asking participants to identify the competition winner from a set of *two* performances rather than from three. In Experiment 3, we used an entirely different set of musical performances and again presented them to participants in pairs rather than triads.

## Experiment 2

### Method

The procedure was identical to Experiment 1, including the use of the Musical Ear Test, with three changes. First, as mentioned above, we chose six-second sections of different performances, obtaining videos from the same musicians in different competitions, or different musicians in the same competitions, or both. Second, to simplify the task, we presented the videos in pairs (i.e., winner and runner-up) rather than in triads. Third, each pair was presented twice before participants indicated their response.

#### Participants and statistical power

We chose a target sample size of 100 per condition (total *N* = 300, age: *M* = 22 years, *SD* = 6.6, range: [15, 64]; sex: 102 male, 198 female), achieving greater than .99 power to detect the effect of size *d* = .58 reported in [1]. Participants were recruited in the same fashion as in Experiment 1.

#### Selection of videos

Using the Supporting Information in [1] as a guide, we selected six second clips of each performance. In an effort to isolate performance quality as the measure of interest, when performers in a given pair played the same piece of music, we selected clips of the same section of the composition. If they played different compositions, we attempted to match the clips on the basis of their style, difficulty, and camera angles. All videos are available at https://osf.io/w9vfw/.

### Results

Performance in both the audio-only and audiovisual conditions was significantly below the chance level of 50% (percent correct, audio-only: *M* = 44.6, *SD* = 13.1, 95% CI: [42.0, 47.2], *d* = 0.41; *t*(99) = 4.11, *p* = .0001; audiovisual: *M* = 42.6, *SD* = 16.1, 95% CI: [39.4, 45.8], *d* = 0.46; *t*(99) = 4.61, *p* < .0001). In contrast to Experiment 1, performance in the visual-only condition was at chance (*M* = 51.1, *SD* = 17.3, 95% CI: [47.7, 54.5]; *t*(99) = 0.64, *p* = .53). As in Experiment 1, musical ability was not related to identification accuracy: in no condition was performance on the Musical Ear Test predictive of performance in identifying the competition winner (from ordinal logistic regressions, *p*s > .4) and when restricting the sample to expert-level performance (greater than 79.8% accuracy, as in Experiment 1), results were similar, with audio-only performance significantly below chance (percent correct, *M* = 44.3, *SD* = 12.4, 95% CI: [39.0, 49.7], *d* = 0.46; *t*(22) = 2.19, *p* = .039), and the other two conditions at chance (audiovisual: *M* = 44.5, *SD* = 17.1, 95% CI: [37.0, 52.1]; *t*(21) = 1.50, *p* = .150; visual-only: *M* = 51.8, *SD* = 15.1, 95% CI: [44.0, 59.5]; *t*(16) = 0.48, *p* = .64). Item analyses revealed that these effects were consistent across trials.

Thus, while performance was slightly higher in the visual than the audio and audio-visual conditions, it was not above chance in the visual condition. These findings show that simple methodological changes (presenting participants with pairs of videos rather than triads and using different portions of the same clips in Tsay, 2013) rendered participants unable to accurately identify competition winners from visual information alone.

### Interim discussion

The results of Experiment 2 demonstrate that the main effect of [1] showing participants’ ability to identify winners in the visual condition, while robustly replicated in Experiment 1, does not hold when different clips from the same performances are used, and when the task is simplified by presenting performances in pairs rather than in triads. We note that altering two aspects of the experiment at once (i.e., presenting videos in pairs rather than triads while also using different clips from the same performances) precludes our ability to isolate the cause of the failure to replicate in Experiment 2; a future experiment presenting only the winner and runner-up performances, in a pair, using the same excerpts as Experiment 1, can tease these explanations apart. However, our goal was to demonstrate that modest design changes cause a failure to replicate, rather than identify the specific changes that do so.

Presenting, moreover, the items in pairs raises a new issue: because chance level is higher in pairs than in triads, a relatively higher rate of performance would be required to detect higher-than-chance performance when using pairs of performances. Arguably, this makes the task more precise. If, in a set of three performances, the lowest-quality is *always* identifiable, participants could guess at random between the remaining two performers, leading to performance with 50% accuracy— easily above the chance level of 33%. A forced choice between two performances has no such issue, and should thus be a more precise measure of participants' true ability to judge performance quality.

Nevertheless, a higher chance level makes it inherently more difficult to detect a true effect (in any of the conditions), should one exist, simply because the range of possible performance levels is restricted. This is especially an issue with effects of modest size. Thus, in Experiment 3, we ran a second conceptual replication where differences in performance quality might be slightly more evident to participants, to determine whether it was possible to detect competition winners in a forced-choice task.

## Experiment 3

### Method

Experiment 3 was identical to Experiment 2, with four changes. First, we used clips of some different performers than those used in [1] (see full listing at https://osf.io/w9vfw/). Second, for brevity, we reduced the number of performer pairs to 5 from 10. Third, given null results in Experiments 1 and 2 vis-à-vis musical ability, we did not collect Musical Ear Test data. Fourth, and most importantly, we paired six-second clips of the winning performers with six-second clips of competitors who were eliminated in earlier rounds of the competition, as opposed to the runners-up that were used in Experiments 1 and 2. Differences in quality across the two performers in each pair should thus be more salient.

#### Participants and statistical power

We chose a target sample size of 50 per condition (total *N* = 150, age: *M* = 19 years, *SD* = 1.3, range: [18, 24]; sex: 46 male, 103 female, 1 chose not to report), achieving power of .98 to detect the effect of size *d* = .58 reported in [1]. Participants were recruited in the same fashion as in Experiment 1.

### Results

Performance differed substantially from the previous two experiments. Performance in both the audio-only and audiovisual conditions was significantly *above* the chance level of 50% (percent correct, audio-only: *M* = 68.4, *SD* = 19.4, 95% CI: [62.9, 73.9], *d* = 0.95, *t*(49) = 6.70, *p* < .0001; audiovisual: *M* = 63.6, *SD* = 19.2, 95% CI: [58.1, 69.1], *d* = .71, *t*(49) = 5.00, *p* < .0001), whereas performance in the visual-only condition was not significantly different than chance (if anything, it trended *below* chance: *M* = 45.2, *SD* = 18.9, 95% CI: [39.8, 50.6], *t*(49) = 1.80, *p* = .078). Between-subjects, performance in the visual-only condition was significantly worse than in the audio-only and audiovisual conditions (*M*_diff_ = 20.8 percentage points, 95% CI: [14.3, 27.3], *t*(101) = 6.31, *p* < .0001; Satterthwaite's *t*-test). Item analyses revealed that these effects were consistent across trials.

## Discussion

In a highly-powered replication of [1] we found that both novice and expert musicians correctly identified the winners of prestigious music competitions in snap judgments from muted videos of the top three finalists, but not when they heard audio-only or viewed and heard audiovisual versions (Experiment 1). Two conceptual replications followed. In the first (Experiment 2), identification of the competition winners dropped to chance when participants viewed a set of comparable muted clips from similar competitions and musicians. Because this experiment altered the design (presenting the videos in triads rather than pairs used in [1]) as well as the materials (the same performances as [1], but different clips from those performances), we cannot isolate the cause of the failure to replicate in Experiment 2. In the second conceptual replication (Experiment 3), we made the task easier by making differences in performance quality likely more salient, and again we failed to replicate: participants successfully identified the competition winners only when they could hear their performances. Across all three experiments, statistical power was very high (above .98).

In sum: Experiments 2 and 3 present very modest changes to the methods of Experiment 1. If the Experiment 1 findings and the original experiment they replicate are robust enough to justify broad conclusions about psychological phenomena (e.g., the evolution of visual dominance; see [1], p. 14583), they should survive minor methodological alterations. They do not.

Our findings call into question the generality and interpretation of the sight-over-sound effects in the judgment of music performance reported in [1] and which we replicated in Experiment 1. If listeners indeed use sight over sound to identify musical expertise, then the conceptual replication in Experiment 2, which used different clips of the same videos from Experiment 1, should have been successful. It was not: in the visual-only condition, participants' identification accuracy was at chance.

Regardless of their musical expertise, most participants surveyed in [1] believed that the characteristic of a musical performance most relevant to evaluating its quality is the sound produced—the music itself, that is—not the way the performer looks while producing it. Consistent with this common-sense belief, in Experiment 3, participants were most able to identify competition winners when they could hear the performances.

Two deflationary accounts should be considered. First, Experiment 2 might have failed because the putative population effect is too small to detect in a two-option forced choice, but large enough to detect when choosing among three videos (whether or not participants' strategy of choosing a winner increases their level of performance in the triad version of the task). Findings in Experiment 3 weigh against this interpretation, however, as in that experiment, the differences in performance quality were even more salient than in Experiment 2, but performance in the visual-only condition trended *below* chance. Further replication of the current results, along with additional conceptual replications using sets of three or four performers, can test this deflationary account.

Second, it is possible that the six-second clips we chose in Experiment 2 had too little variance in performance quality to detect the winner; e.g., in a technically facile portion of the performance. With limited time and variance in performance quality, even the most sensitive listener might have insufficient information to detect the superior performance. Without a content analysis of the clips themselves, we cannot rule out this deflationary account. However, the issue cuts both ways: it is also possible that the 6-second clips in [1] were selected on the basis of more visual flair from the performer, or even from visual factors unrelated to the performer, such as the camera angle, the number of visible performers or audience members, the enthusiasm of both groups of people, and so on; any of these factors could introduce confirmation bias.

A review of the videos used in [1] and in our Experiment 1, which we encourage readers to watch and listen to (see https://osf.io/w9vfw/), reveals a wide variety of differences between videos within and across triads: some focus in great detail on the performer's physical interaction with the musical instrument, while others reveal no such information, as the performer's hands are obscured; some performers wear revealing outfits while being compared to others that are more conservatively dressed; and some use a static camera while others are filmed in motion. Thus, it is difficult to conclude that high performance in the visual-only condition is due to gesturally conveyed expressiveness. This same issue is present in a conceptual replication of [1] using six-second clips of orchestral performances, with similar findings [7]. A more rigorous test of this proposal should present excerpts matched for the degree of motion across performers, excerpts filmed from identical angles or with extraneous visual information obscured, or excerpts where the degree of visual flair is experimentally manipulated. We predict that if different six-second clips were judged from the same performances, selected with one of the methods above, the results will not hold.

It is now well known that surprising and counter-intuitive results require replication (e.g., [2–3]). The present findings demonstrate the value of both direct and conceptual replications: whereas Experiment 1 shows that the previously reported sight-over-sound effect replicated when the same video clips were used, findings of interest must generalize beyond the materials used in the original test. Direct replications cannot test such generalization, but conceptual replications can.

We have shown a failure to replicate a previous report of sight trumping sound in the evaluation of music performances [1] when we made modest changes to materials and methods (e.g., some different musicians and competitions in Experiment 2; clearer differences in performance quality in Experiment 3; presenting the performances in pairs rather than triads in both experiments). Sight-over-sound effects in the judgment of music performances [1] taken together with the much less counter-intuitive results that we report here, may thus provide a case study for improving the reliability of psychological science.
